# Influence of tumor thrombus morphology on the surgical complexity in renal cell carcinoma with inferior vena cava tumor thrombus: a single-center, large-sample study from China

**DOI:** 10.1007/s00345-024-05170-3

**Published:** 2024-07-29

**Authors:** Xun Zhao, Zhuo Liu, Ji-yuan Chen, Wei Guo, Hong-xian Zhang, Xiao-jun Tian, Guo-liang Wang, Cheng Liu, Lu-lin Ma, Shu-dong Zhang

**Affiliations:** 1 Department of Urology, Peking University Third Hospital, 49 Huayuan North Road, Haidian District, Beijing, 100191 China; 2 Department of Urology, Yan’an Hospital of Traditional Chinese Medicine, 26 Xuanyuan Road, Bridge Ditch Street, Baota District, Yan’an, Shanxi Province 716000 China

**Keywords:** Tumor thrombus morphology, Renal cell carcinoma, Inferior Vena cava tumor thrombus, Surgical complexity

## Abstract

**Background:**

The morphology of tumor thrombus varies from person to person and it may affect surgical methods and tumor prognosis. However, studies on the morphology of tumor thrombus are limited. The purpose of our study was to evaluate the impact of tumor thrombus morphology on surgical complexity.

**Methods:**

We retrospectively reviewed the clinical data of 229 patients with renal cell carcinoma combined with inferior vena cava (IVC) tumor thrombus who underwent surgical treatment at Peking University Third Hospital between January 2014 and December 2021. The patients were divided into floating morphology (107 patients) and filled morphology (122 patients) tumor thrombi groups. Chi-square and Mann–Whitney U tests were used for categorical and continuous variables, respectively. Postoperative complications were evaluated using the Clavien–Dindo surgical complication classification method.

**Results:**

Patients with filled morphology tumor thrombus required more surgical techniques than those with floating morphology tumor thrombus, which was reflected in more open surgeries (*P* < 0.001), more IVC interruptions (*P* <0.001), lesser use of the delayed occlusion of the proximal inferior vena cava (DOPI) technique (*P* < 0.001), and a greater need for cut-off of the short hepatic vein (*P* < 0.001) and liver dissociation (*P* = 0.001). Filled morphology significantly increased the difficulty of surgery in patients with renal cell carcinoma with tumor thrombus, reflected in longer operation time (*P* < 0.001), more surgical blood loss (*P* <0.001), more intra-operative blood transfusion (*P* < 0.001), and longer postoperative hospital stay (*P* < 0.001). Filled morphology tumor thrombus also led to more postoperative complications (53% vs. 20%; *P* < 0.001).

**Conclusion:**

Compared with floating morphology thrombus, filled morphology thrombus significantly increased the difficulty of surgery in patients with renal cell carcinoma with IVC tumor thrombus.

**Supplementary Information:**

The online version contains supplementary material available at 10.1007/s00345-024-05170-3.

## Introduction

Renal cell carcinoma (RCC) is a common malignancy of the urinary system, accounting for 2–3% of adult malignant tumors [1]. An essential clinical feature of RCC is that it often invades the renal vein and inferior vena cava (IVC) and forms a venous tumor thrombus in approximately 4–10% of patients with RCC [2]. Radical nephrectomy and thrombectomy is the only possible radical treatment. After complete removal of the tumor and tumor thrombus, a 5-year survival rate of more than 50% can be achieved, whereas the 5-year survival rate is only approximately 10% when the resection is incomplete [3]. Clarifying the growth characteristics of tumor thrombus has essential clinical value in diagnosing and treating the various types of tumor thrombus. In clinical practice, the morphology of tumor thrombus varies from person to person, with some thrombi floating in the veins (floating morphology), while others fill the entire IVC (filled morphology). Tumor thrombus morphology may affect surgical methods and tumor prognosis. However, studies on the morphology of tumor thrombus are limited. The purpose of this study was to evaluate the impact of tumor thrombus morphology on the surgical complexity and prognosis of patients with renal cell carcinoma with venous tumor thrombus.

## Patients and methods

### Patients

We retrospectively analyzed the clinical data of patients with RCC with IVC tumor thrombus who were admitted to the Department of Urology, Peking University Third Hospital between January 2014 and December 2021. The inclusion criteria were as follows: (a) preoperative examination confirmed a renal mass with IVC tumor thrombus, (b) patients who underwent radical nephrectomy and thrombectomy, and (c) postoperative pathology was RCC. The exclusion criteria were as follows: (a) non-operation, (b) bilateral tumor with thrombus, (c) recurrent tumor with thrombus, (d) Mayo grade 0 tumor thrombus, and (e) incomplete clinical data. In total, 379 nephrectomies and thrombectomies were performed between 2014 and 2021. After exclusion, 229 patients were included in this study (Supplementary material 1). In this study, 197 (86.0%) patients underwent surgery performed by four experienced chief physicians, and 32 (14.0%) patients underwent surgery performed by eight experienced deputy chief physicians. All surgeons had extensive experience as the main surgeon or assistant in nephrectomy and thrombectomy prior to this study.

In this study, 107 (46.7%) and 122 (53.3%) patients were classified as having a floating morphology thrombus and filled morphology thrombus, respectively. The criterion was as follows: on the transverse plane of enhanced CT or MRI, if there was a plane where the tumor thrombus completely filled the IVC and the gap between the tumor thrombus and wall of the IVC was less than 1 mm, the thrombus was called a filled tumor thrombus. If there were gaps in all planes, the thrombus was called a floating tumor thrombus (Supplementary material 2). During surgery, IVC with floating morphology thrombus was not significantly thickened, and blue venous blood flow could be seen inside. After the vein was blocked, the IVC was partially flattened and the blood flow disappeared. When the IVC wall was cut, there was a gap between the vein wall and tumor thrombus. In contrast, IVC with filled morphology thrombus was significantly thickened, with no obvious blood flow. The width of IVC did not decrease significantly after occlusion, the space between the vein wall and tumor thrombus was not obvious after incision of the IVC, and the probability of adhesion between the tumor thrombus and IVC wall increased (Supplementary material 3).

The perioperative clinical data were also collected. The tumor thrombus is divided into five categories according to the position of the proximal end of the tumor thrombus using the Mayo classification [4]. A GADVR thrombus was defined as a tumor thrombus growing against the direction of venous return [5]. Bland thrombus was diagnosed using MRI, and the diagnostic criteria were based on our previous studies [6]. A bland thrombus is a non-tumor thrombus, mainly composed of activated platelets, macrophages, and fibrin.

The surgical methods were divided into laparoscopic minimally invasive approach, robot-assisted laparoscopy, and open approach. The surgical approach followed in our institution has been previously described [7, 8]. IVC interruption was used when a tumor thrombus invaded the vessel wall extensively or when there was an extended bland thrombus at the distal end of a tumor thrombus [9]. Diagnostic imaging methods for invasion of the IVC wall have been described in previous studies [10]. The delayed occlusion of the proximal IVC (DOPI) technique was applied in some patients to simplify the procedures of dissociating and exposing the proximal IVC (only applied in patients with right RCC) [11]. Foley catheter-assisted thrombectomy was performed in some patients to avoid thoracotomy or cardiopulmonary bypass [12]. Postoperative complications were evaluated using the Clavien–Dindo surgical complication classification method, in which grades ≥ 3 were defined as severe complications [13].

All patients were followed up once every 6 months until year 5 and once every year thereafter. All patients were followed-up in the outpatient clinic or by telephone to obtain prognostic information.

### Statistical analysis

Continuous variables are shown as medians (Q1, Q3) and were analyzed using the Mann–Whitney U test. Categorical variables were summarized as percentages, and a chi-square test was performed. Kaplan–Meier plots were used to evaluate the influence of thrombus morphology on overall survival (OS). SPSS (version 18.0, IBM Corporation, USA) was used for statistical analysis, and *P* < 0.05 indicated that the difference was statistically significant. Survival time was defined as the time from the date of surgery to the date of death or last follow-up.

## Results

The clinical and pathological features of all patients are shown in Supplementary material 4. There were no significant differences in age, sex, pathological morphology, or other baseline characteristics between the groups. Compared with the floating morphology tumor thrombus group, the filled morphology tumor thrombus group had statistically larger maximum width of tumor thrombus (27.2 mm vs. 19.7 mm, *P* < 0.001), larger width of tumor thrombus at the entrance of the renal vein (16.9 mm vs. 15.5 mm, *P* = 0.016), higher Mayo classification (*P* < 0.001), a higher proportion of bland thrombus (*P* = 0.027), a higher proportion of GADVR thrombus (*P* = 0.010), and higher WHO/ISUP nuclear grade (*P* = 0.049). In general, filled morphology tumor thrombus was higher and wider, resulting in blood flow obstruction (reflected in a higher proportion of bland and GADVR thrombi).

The surgical features of all patients are shown in Table 1. In terms of the influence on surgery, patients with filled morphology tumor thrombus had longer operation time (*P* < 0.001), more surgical blood loss (*P* <0.001), more intra-operative blood transfusions (*P* < 0.001), more plasma transfusions (*P* < 0.001), more IVC interruptions (P  <0.001), lesser use of DOPI technique (P  <0.001), a greater need for cut-off of the short hepatic vein (*P* < 0.001) and liver dissociation (*P* = 0.001), and longer postoperative hospital stay (POHS) (*P* < 0.001) than patients with floating morphology tumor thrombus. Filled morphology tumor thrombus also led to more postoperative complications (53% vs. 20%; *P* < 0.001). Regarding the surgical method, patients with filled morphology tumor thrombus had a higher proportion of open surgeries than those with floating morphology tumor thrombus (63.1% vs. 32.7%, *P* < 0.001). There was no case of robotic surgery conversion to open surgery. For laparoscopic surgery, the conversion rate to open surgery was 37.8% for filled morphology (17/45) and 19.4% for floating morphology (14/72). Hence, filled morphology increases the difficulty of nephrectomy and thrombectomy. However, there was no difference in the incidence of severe complications between patients with filled morphology and floating morphology thrombi, indicating that surgical treatment of filled morphology tumor thrombus is safe and reliable.

**Table 1:** Comparison of surgical features between floating morphology and filled morphology tumor thrombus

Surgical characteristic	Floating morphology(*N* = 107)	Filled morphology(*N* = 122)	*P*
Operative time, min	309(230,399)	370(301,444)	< 0.001
Surgical blood loss, ml	550(200,1200)	1200(500,2525)	< 0.001
Intra-operative blood transfusion, ml	0(0,800)	800(400,1600)	< 0.001
Intra-operative plasma transfusion, ml	0(0,200)	400(0,600)	< 0.001
Postoperative hospital stay, days	8(6,10)	10(7,13)	< 0.001
Surgical approach			< 0.001
Completely Laparoscopic	47(43.9%)	24(19.7%)	
Robot-assisted laparoscopic	11(10.3%)	4(3.3%)	
Open	35(32.7%)	77(63.1%)	
Conversion of laparoscopy to open surgery	14(13.1%)	17(13.9%)	
DOPI technique	46(43.0%)	14(11.5%)	< 0.001
IVC interruption	6(5.6%)	37(30.3%)	< 0.001
Incision of diaphragm	2(1.9%)	5(4.1%)	0.453
Open thoracotomy	3(2.8%)	4(3.3%)	0.835
Cut-off of the short hepatic veins	26(24.3%)	75(61.5%)	< 0.001
Liver dissociation	14(13.1%)	38(31.1%)	0.001
Extracorporeal circulation	3(2.8%)	3(2.5%)	0.871
Foley catheter-assisted	9(8.4%)	18(14.8%)	0.155
Transesophageal ultrasound	8(7.5%)	20(16.4%)	0.045
Overall complications	21(20%)	61(53%)	< 0.001
Severe complications	5(4.7%)	10(8.2%)	0.423

DOPI, delayed occlusion of the proximal inferior vena cava; IVC, inferior vena cava

The Mayo classification is crucial for assessing the difficulty of surgery. Due to the higher Mayo classification of tumor thrombus in the filled morphology group, we conducted a subgroup analysis of Mayo level II tumor thrombi in order to further independently evaluate the impact of thrombus morphology on surgical difficulty (Supplementary material 5). For Mayo level II tumor thrombi, the results were similar. Patients with filled morphology tumor thrombus had longer operation time (*P* = 0.009), more surgical blood loss (*P* =0.002), more intra-operative blood transfusions (*P* < 0.001), more plasma transfusions (*P* < 0.001), more IVC interruptions (*P* = 0.003), a greater need for cut-off of the short hepatic vein (*P* = 0.009), and longer POHS (*P* = 0.021) than those with floating morphology. Filled tumor thrombus morphology also led to more open surgeries (55.6% vs. 28.3%, *P* = 0.016) and postoperative complications (54.4% vs. 28.9%, *P* = 0.012).

**Fig. 1:**
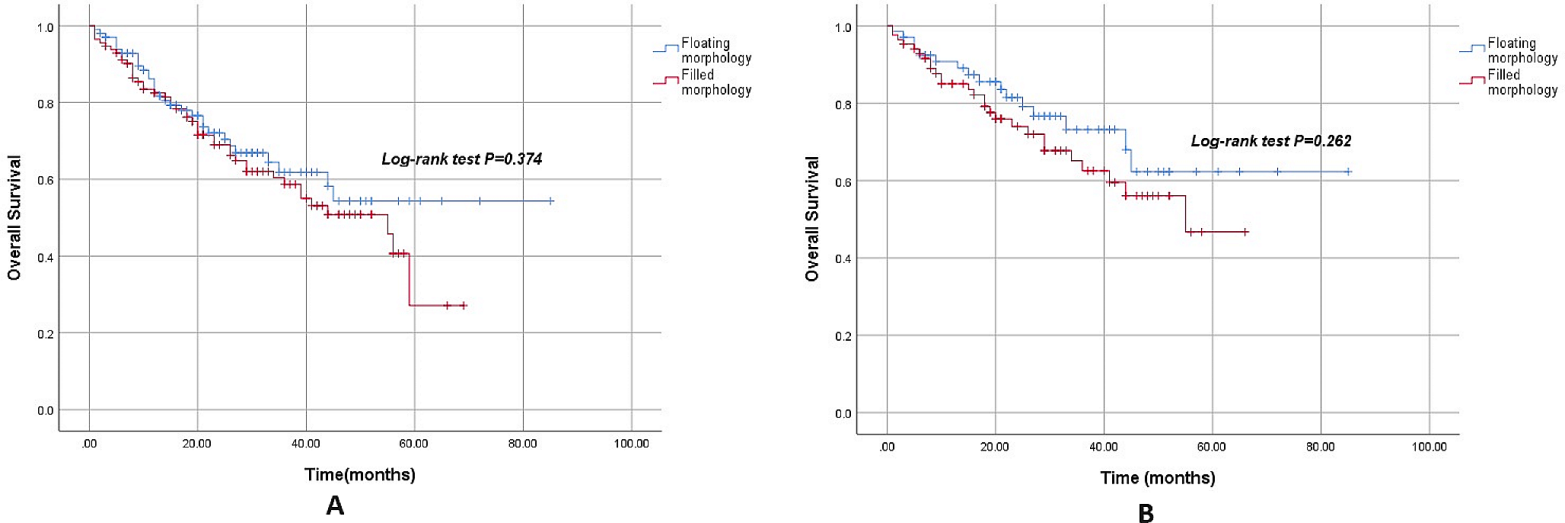
**A:** Overall survival of patients with filled morphology and floating morphology tumor thrombi. **B:** Overall survival of filled morphology and floating morphology tumor thrombi in cM0 subgroup

The median follow-up time was 21.0 months (1.0–85.0 months). There was no statistical difference in OS between patients with filled morphology and floating morphology tumor thrombi (42.2 ± 2.8 months vs. 55.8 ± 4.2 months, *P* = 0.374) (Fig. 1A). In the subgroup of non-metastatic renal cell carcinoma with tumor thrombus, there was no statistical difference (45.2 ± 3.1 months vs. 62.3 ± 4.8 months, *P* = 0.262) (Fig. 1B).

## Discussion

The growth characteristics of tumor thrombus are still unclear. Some tumor thrombi grow along the direction of blood flow (longitudinal growth) and float in the IVC, whereas others grow laterally and fill the entire IVC, resulting in blood flow obstruction. We divided tumor thrombi into two morphologies to explore its impact. At present, the height of the tumor thrombus is considered before thrombectomy, and the influence of the width remains unclear. Due to individual differences in the width of the IVC, the width of tumor thrombus is easily influenced by individual differences. Therefore, we believe that the morphology of tumor thrombus, rather than the width, can better reflect the characteristics of the tumor. Based on whether the tumor thrombus filled the entire IVC, we classified tumor thrombus into two morphologies: filled and floating. These criteria were based on enhanced CT/MRI. In general, a filled morphology tumor thrombus results in blood flow obstruction, reflected in a higher proportion of bland and GADVR thrombi. Previous studies have shown that a filled morphology tumor thrombus is more likely to invade the venous wall [14]. These findings indicate that the morphology of the tumor thrombus may affect the operation and prognosis.

Radical nephrectomy and thrombectomy can effectively improve the prognosis of patients with RCC with IVC tumor thrombus. The purpose of surgical treatment is to extensively remove the tumor burden. However, filled morphology significantly increased the difficulty of complete removal of the tumor thrombus. In our study, surgeries in patients with filled morphology tumor thrombus were much more difficult than in those with floating morphology tumor thrombus, which was reflected in longer operation time, more surgical blood loss, more intra-operative blood transfusions, more open surgeries, and more IVC interruptions. Correspondingly, recovery of patients with filled morphology tumor thrombus was more difficult, with longer hospital stays and more postoperative complications. Chung et al. found that racial factors were associated with a markedly elevated rate of major complications [15]; however, the population included in our study was all Chinese, avoiding the influence of racial factors.

The increase in surgical difficulty is consistent with our clinical experience and previous research [16]. The reasons for this might be as follows. (A) The proportion of high-level tumor thrombus was higher in the filled morphology thrombus group, with levels III–IV accounting for 33.6%, whereas it was 12.1% in the floating morphology group. The surgical difficulty in patients with high-level tumor thrombus was higher than that of those with low-level tumor thrombus. Therefore, we conducted a subgroup analysis of Mayo level II tumor thrombus to further independently evaluate the impact of thrombus morphology on surgical difficulty and the results were similar, which means that the impact of tumor thrombus morphology on surgical difficulty is independent of the Mayo classification. (B) Filled morphology leads to obstruction of the IVC blood flow, which is more likely to form long-segment bland thrombus and GAVDR thrombus. Moreover, it is easier to invade the wall of the IVC. This makes it difficult to remove the tumor thrombus. Interruption of the IVC is occasionally necessary to achieve basic oncological control. Previous studies have reported that approximately 6–8% of patients require IVC interruption [11]. In our study, IVC interruption was performed in 30.3% of patients with filled morphology tumor thrombus but only in 5.6% of patients with floating morphology tumor thrombus. (C) Some surgical techniques that can simplify the operation are more suitable for floating morphology tumor thrombus, such as the DOPI technique (43% vs. 11.5%). (D) Filled morphology may represent a more aggressive tumor subtype that requires more time for tumor dissociation during surgery.

A systematic review showed that a large tumor size, high Fuhrman grade, tumor necrosis, positive lymph nodes, and metastasis were significant adverse predictors of OS in patients with RCC and IVC tumor thrombus [17]. In this study, the mean survival time of patients with filled morphology thrombus was 42.2 months, while that of patients with floating morphology thrombus was 55.8 months, although the difference was not significant. Previous studies have shown that IVC wall invasion, bland thrombus, and GAVDR thrombus are independent prognostic factors of RCC with tumor thrombus [5, 6, 16]. However, our study showed that the morphology of the tumor thrombus does not affect the prognosis. The reasons might be as follows: (A) The number of patients and follow-up time were insufficient; hence, although the impact on prognosis had only one trend, it did not reach statistical significance. (B) The filled morphology was not enough to lead to a poor prognosis, which means that tumor thrombus simply filling the IVC is not sufficient, but it must also cause invasion of the IVC wall, grow against the direction of venous return, form a bland thrombus, and cause other changes to lead to a poor prognosis. However, the specific reasons for this need to be confirmed through further research.

This study had some limitations. Although the data were obtained from one of the largest RCC and IVC tumor thrombus sample centers in China, there may be uncontrollable confounding factors and inherent selection bias. The short follow-up period was also a limitation of this study. Surgical methods are crucial to assess the difficulty of a surgery, which is an important limitation of this study. However, the surgical method itself is influenced by the tumor thrombus morphology, which may be a reason for thrombus morphology to affect the difficulty of surgery. In addition, this was a single-center retrospective study with a limited follow-up time, and future multi-center prospective studies need to be designed for deeper pathological and genetic analyses.

## Conclusions

In summary, compared with floating morphology thrombus, a filled morphology thrombus significantly increased the difficulty of surgery in patients with renal cell carcinoma with IVC tumor thrombus.

## Electronic supplementary material

Below is the link to the electronic supplementary material.

Supplementary Material 1

Supplementary Material 2

Supplementary Material 3

Supplementary Material 4

Supplementary Material 5

## Data Availability

The datasets used and/or analyzed in the current study are available from the corresponding author upon reasonable request.

## Abbreviations

RCC: Renal cell carcinoma
TT: Tumor thrombus
IVC: Inferior vena cava
OS: Overall survival
GADVR: Growing against the direction of venous return
ASA: American Society of Anesthesiologists
