# Supplementary figures and images for: Influence of tumor thrombus morphology on the surgical complexity in renal cell carcinoma with inferior vena cava tumor thrombus: a single-center, large-sample study from China
Source: World J Urol. 2024 Jul 29;42(1):454. doi: 10.1007/s00345-024-05170-3 (PMC11286623; doi:10.1007/s00345-024-05170-3)

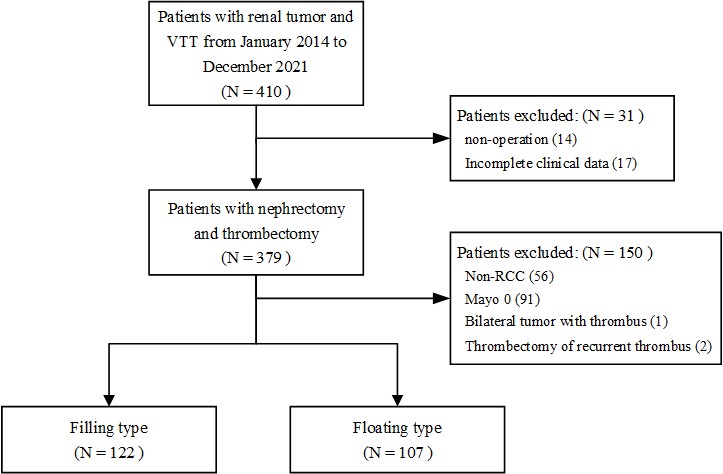


Supplementary material 1: Flowchart of the study. VTT, venous tumor thrombus.

Supplement: Supplementary file 1 — Supplementary Material 1 [file 345_2024_5170_MOESM1_ESM.docx]
