# Supplementary material for: Influence of tumor thrombus morphology on the surgical complexity in renal cell carcinoma with inferior vena cava tumor thrombus: a single-center, large-sample study from China
Source: World J Urol. 2024 Jul 29;42(1):454. doi: 10.1007/s00345-024-05170-3 (PMC11286623; doi:10.1007/s00345-024-05170-3)
Supplement: Supplementary file 3 — Supplementary Material 3 [file 345_2024_5170_MOESM3_ESM.docx]

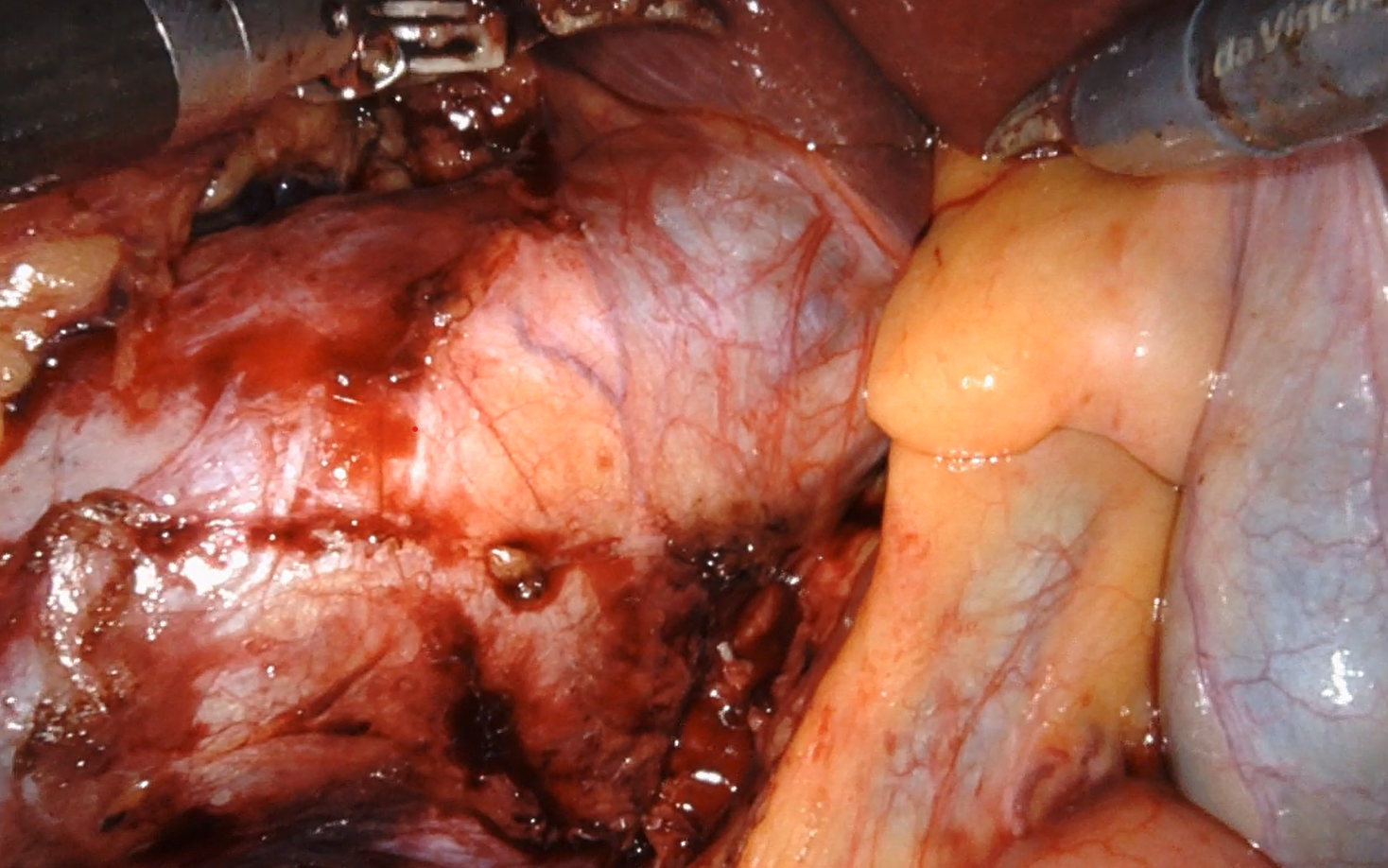

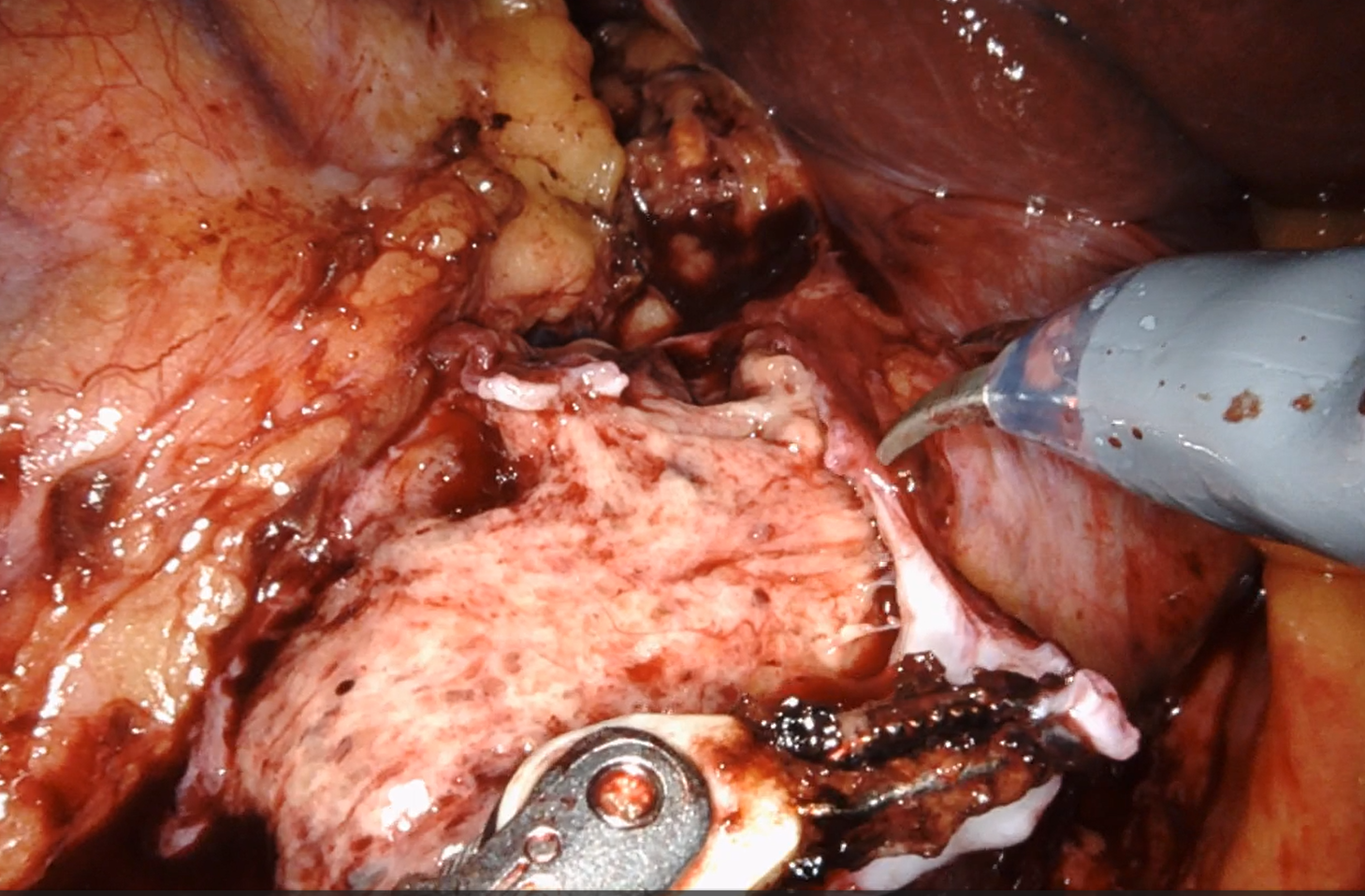

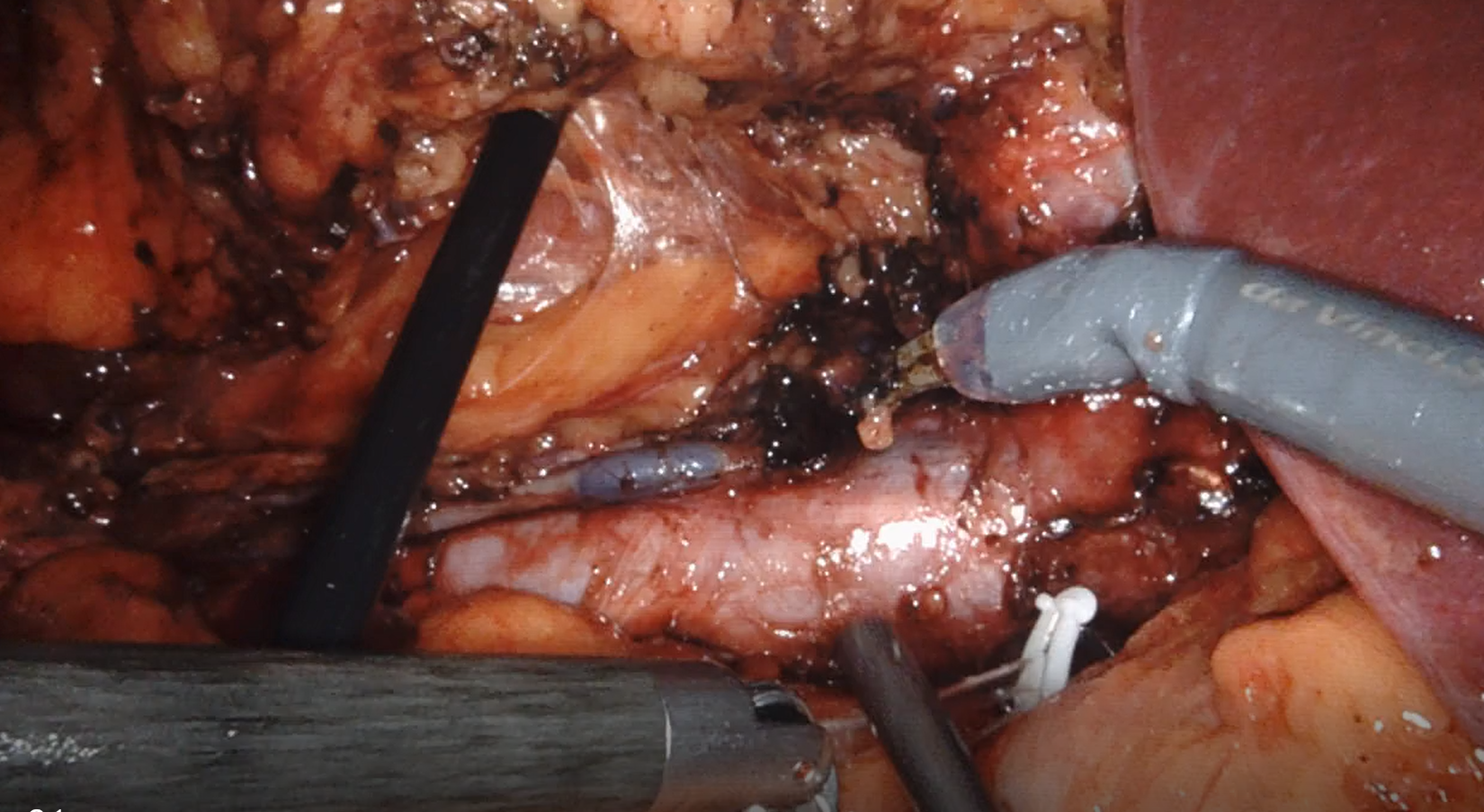

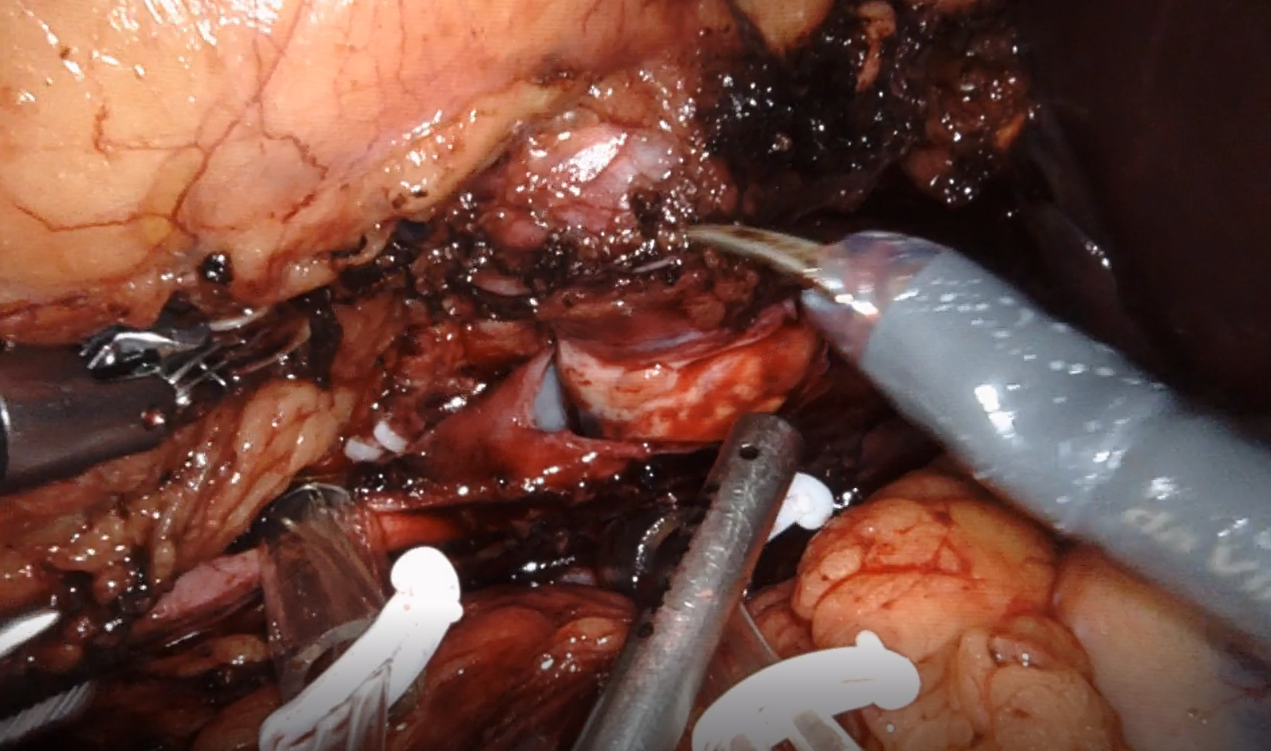


Supplementary material 3: intra-operative findings in typical patients. A: Right RCC with a filled morphology tumor thrombus in the IVC. A significantly thickened IVC can be seen during surgery. B: Right RCC with a filled morphology tumor thrombus in the IVC. No obvious space is present between the IVC and tumor thrombus. C: Right RCC with a floating morphology tumor thrombus in the IVC. Blue venous blood flow was observed during surgery. D: Right RCC with a floating morphology tumor thrombus in the IVC. An obvious space is seen between the IVC and tumor thrombus. IVC, inferior vena cava; RCC, renal cell carcinoma.
