# Supplementary material for: Influence of tumor thrombus morphology on the surgical complexity in renal cell carcinoma with inferior vena cava tumor thrombus: a single-center, large-sample study from China
Source: World J Urol. 2024 Jul 29;42(1):454. doi: 10.1007/s00345-024-05170-3 (PMC11286623; doi:10.1007/s00345-024-05170-3)
Supplement: Supplementary file 4 — Supplementary Material 4 [file 345_2024_5170_MOESM4_ESM.docx]

Table: Comparison of clinical and pathologic features between Floating morphology and Filled morphology.

| Clinicopathological characteristic | Floating morphology(N=107) | Filled morphology(N=122) | P |
| --- | --- | --- | --- |
| Age, years | 60(54,68) | 61(53.8,67.2) | 0.582 |
| BMI, kg/m² | 23.4(21.0,25.8) | 23.9(21.4,26.3) | 0.542 |
| Tumor diameter, cm | 8.7(6.6,10.7) | 8.4(6.5,10.5) | 0.538 |
| Maximum width of TT, mm | 19.7(15.3,23.3) | 27.2(22.8,31.9) | <0.001 |
| The width of TT at the entrance of the renal vein, mm | 15.5(10.4,18.3) | 16.9(13.3,19.6) | 0.016 |
| Hemoglobin, g/L | 125(109,140) | 119(104,129) | 0.047 |
| Platelet count, ×10^9^/L | 247(190,321) | 222(178,286) | 0.058 |
| Neutrophils counts, ×10^9^/L | 4.54(3.50,5.76) | 4.46(3.41,5.56) | 0.659 |
| Serum calcium, mg/dl | 2.27(2.17,2.36) | 2.30(2.18,2.39) | 0.302 |
| Albumin, g/L | 39.9(34.5,42.0) | 39(35.8,43.0) | 0.774 |
| Alkaline phosphatase, U/L | 93.0(73.5,113.5) | 84.0(68.9,103.8) | 0.089 |
| Preoperative serum creatinine, μmol/L | 89.0(78.5,107.0) | 99.0(82.5,113.5) | 0.034 |
| Serum creatine 1 week after operation, μmol/L | 104.5(88.8,125.0) | 99.5(79.0,123.3) | 0.39 |
| Gender |  |  | 0.184 |
| Male | 82(76.6%) | 83(68.0%) |  |
| Female | 25(23.4%) | 39(32.0%) |  |
| Side |  |  | 0.307 |
| Left | 34(31.8%) | 31(25.4%) |  |
| Right | 73(68.2%) | 91(74.6%) |  |
| ASA grade |  |  | 0.604 |
| I-II | 90(84.1%) | 99(81.1%) |  |
| III-IV | 17(15.9%) | 23(18.9%) |  |
| Clinical symptoms |  |  | 0.908 |
| No clinical symptoms | 19(17.8%) | 26(21.3%) |  |
| Local symptoms | 59(55.1%) | 66(54.1%) |  |
| Systemic symptoms | 10(9.3%) | 11(9.0%) |  |
| Both | 19(17.8%) | 19(15.6%) |  |
| cN stage |  |  | 0.317 |
| cN0 | 37(34.6%) | 34(27.9%) |  |
| cN1 | 70(65.4%) | 88(72.1%) |  |
| cM stage |  |  | 0.472 |
| cM0 | 72(67.3%) | 88(72.1%) |  |
| cM1 | 35(32.7%) | 34(27.9%) |  |
| Mayo classification |  |  | <0.001 |
| I | 48(44.9%) | 9(7.4%) |  |
| II | 46(43.0%) | 72(59%) |  |
| III | 6(5.6%) | 23(18.9%) |  |
| IV | 7(6.5%) | 18(14.8%) |  |
| Bland thrombus |  |  | 0.027 |
| No | 90(84.1%) | 87(71.3%) |  |
| Yes | 17(15.9%) | 35(28.7%) |  |
| Perirenal fat invasion |  |  | 0.561 |
| No | 74(69.2%) | 89(73.0%) |  |
| Yes | 33(30.8%) | 33(27.0%) |  |
| Ipsilateral adrenal gland invasion |  |  | 0.656 |
| No | 98(91.6%) | 109(89.3%) |  |
| Yes | 9(8.4%) | 13(10.7%) |  |
| GADVR |  |  | 0.010 |
| No | 88(82.2%) | 82(67.2%) |  |
| Yes | 19(17.8%) | 40(32.8%) |  |
| Pathology morphology |  |  | 0.413 |
| Clear cell RCC | 88(82.2%) | 94(77.0%) |  |
| Non-clear cell RCC | 19(17.8%) | 28(23.0%) |  |
| WHO/ISUP nuclear grade |  |  | 0.049 |
| I-II | 43(40.2%) | 33(27.0%) |  |
| III-IV | 64(59.8%) | 89(73.0%) |  |
| Sarcomatoid differentiation |  |  | 0.147 |
| No | 86(80.4%) | 107(87.7%) |  |
| Yes | 21(19.6%) | 15(12.3%) |  |
