# Supplementary material for: Influence of tumor thrombus morphology on the surgical complexity in renal cell carcinoma with inferior vena cava tumor thrombus: a single-center, large-sample study from China
Source: World J Urol. 2024 Jul 29;42(1):454. doi: 10.1007/s00345-024-05170-3 (PMC11286623; doi:10.1007/s00345-024-05170-3)
Supplement: Supplementary file 5 — Supplementary Material 5 [file 345_2024_5170_MOESM5_ESM.docx]

Table: Comparison of surgical features between Floating morphology and Filled morphology in Mayo II level tumor thrombus subgroup.

| Surgical characteristic | Floating morphology(N=46) | Filled morphology(N=72) | P |
| --- | --- | --- | --- |
| Operative time, min | 298.5(227.0,387.0) | 362.0(292.8,453.3) | 0.009 |
| Surgical blood loss, ml | 600(200,1000) | 1100(433,2650) | 0.002 |
| Intra-operative blood transfusion, ml | 0(0,800) | 800(400,1700) | <0.001 |
| Intra-operative plasma transfusion, ml | 0(0,0) | 400(0,800) | <0.001 |
| Postoperative hospital stay, days | 8(6,12) | 10(7,13) | 0.021 |
| Surgical approach |  |  | 0.016 |
| Completely Laparoscope | 16(34.8%) | 17(23.6%) |  |
| Robot-assisted laparoscope | 7(15.2%) | 3(4.2%) |  |
| Open | 13(28.3%) | 40(55.6%) |  |
| Laparoscope conversion to open surgery | 10(21.7%) | 12(16.7%) |  |
| DOPI technique | 11(23.9%) | 9(12.5%) | 0.133 |
| IVC interruption | 4(8.7%) | 24(33.3%) | 0.003 |
| Incision of diaphragm | 0(0%) | 0(0%) | - |
| Open thoracotomy | 0(0%) | 0(0%) | - |
| Cut-off of short hepatic veins | 14(30.4%) | 40(55.6%) | 0.009 |
| Liver dissociation, | 5(10.9%) | 11(15.3%) | 0.589 |
| Extracorporeal circulation, | 0(0%) | 0(0%） | - |
| Foley catheter-assisted | 1(2.2%) | 4(5.6%) | 0.647 |
| Transesophageal ultrasound | 2(4.3%) | 4(5.6%) | 1.000 |
| Overall complications | 13(28.9%) | 37(54.4%) | 0.012 |
| severe complications | 3(6.5%) | 5(6.9%) | 1.000 |
